# Isovaline monohydrate

**DOI:** 10.1107/S1600536813031620

**Published:** 2013-11-27

**Authors:** Ray J. Butcher, Greg Brewer, Aaron S. Burton, Jason P. Dworkin

**Affiliations:** a Department of Chemistry, Howard University, 525 College Street NW, Washington, DC 20059, USA; b Department of Chemistry, Catholic University of America, Washington, DC 20064, USA; c NASA Johnson Space Center, Astromaterial and Exploration Science Directorate, Houston, TX 77058, USA; d Solar System Exploration Division, NASA Goddard Space Flight Center, Greenbelt, MD 20771, USA

## Abstract

The title compound, C_5_H_11_NO_2_·H_2_O, is an isomer of the α-amino acid valine that crystallizes from water in its zwitterion form as a monohydrate. It is not one of the 20 proteinogenic amino acids that are used in living systems and differs from the natural amino acids in that it has no α-H atom. The compound exhibits hydrogen bonding between the water mol­ecule and the carboxyl­ate O atoms and an amine H atom. In addition, there are inter­molecular hydrogen-bonding inter­actions between the carboxyl­ate O atoms and amine H atoms. In the crystal, these extensive N—H⋯O and O—H⋯O hydrogen bonds lead to the formation of a three-dimensional network.

## Related literature

The structure of the title compound or its salts have not been reported to the CCDC but there are reports of homoleptic coordination complexes of zinc(II) with isovaline, see: Strasdelt *et al.* (2001 ▶). For literature related to eighty amino acids that have been detected in meteorites or comets, see: Glavin & Dworkin (2009 ▶); Burton *et al.* (2012 ▶). For the role that crystallization plays in chiral separation, see: Blackmond & Klussmann (2007 ▶); Blackmond *et al.* (2008 ▶). For the role of the H atom on the α-C atom in enhancing the rate of racemization, see: Yamada *et al.* (1983 ▶). For the mechanism of racemization of amino acids lacking an α-H atom, see: Pizzarello & Groy (2011 ▶). For the role that crystallization can play in the enrichment of l-isovaline, see: Glavin & Dworkin (2009 ▶); Bada (2009 ▶); Bonner *et al.* (1979 ▶). For normal bond lengths and angles, see: Orpen (1993 ▶).
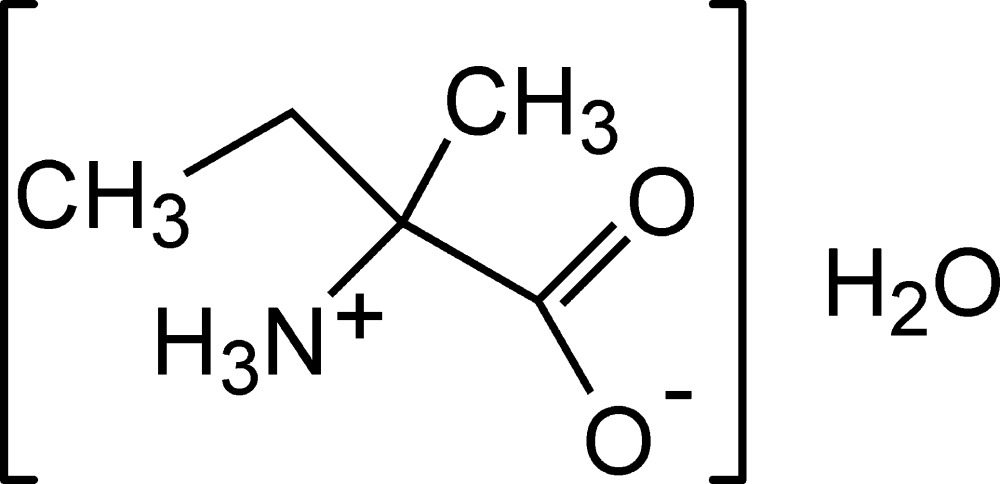


## Experimental

### 

#### Crystal data

C_5_H_11_NO_2_·H_2_O
*M* *_r_* = 135.16
Orthorhombic, 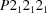

*a* = 5.9089 (5) Å
*b* = 10.4444 (10) Å
*c* = 11.9274 (11) Å
*V* = 736.10 (12) Å^3^
*Z* = 4
Cu *K*α radiation
μ = 0.84 mm^−1^
*T* = 123 K
0.48 × 0.08 × 0.06 mm

#### Data collection

Agilent Xcalibur (Ruby, Gemini) diffractometer
Absorption correction: multi-scan (*CrysAlis PRO*; Agilent, 2012 ▶) *T*_min_ = 0.383, *T*_max_ = 1.000
1662 measured reflections
1204 independent reflections
1072 reflections with *I* > 2σ(*I*)
*R*_int_ = 0.072

#### Refinement

*R*[*F* ^2^ > 2σ(*F* ^2^)] = 0.056
*wR*(*F* ^2^) = 0.162
*S* = 1.11
1204 reflections
91 parameters
3 restraints
H atoms treated by a mixture of independent and constrained refinement
Δρ_max_ = 0.37 e Å^−3^
Δρ_min_ = −0.27 e Å^−3^

### 

Data collection: *CrysAlis PRO* (Agilent, 2012 ▶); cell refinement: *CrysAlis PRO*; data reduction: *CrysAlis PRO*; program(s) used to solve structure: *SHELXS97* (Sheldrick, 2008 ▶); program(s) used to refine structure: *SHELXL97* (Sheldrick, 2008 ▶); molecular graphics: *SHELXTL* (Sheldrick, 2008 ▶); software used to prepare material for publication: *SHELXTL*.

## Supplementary Material

Crystal structure: contains datablock(s) I. DOI: 10.1107/S1600536813031620/jj2178sup1.cif

Structure factors: contains datablock(s) I. DOI: 10.1107/S1600536813031620/jj2178Isup2.hkl

Supplementary material file. DOI: 10.1107/S1600536813031620/jj2178Isup3.cml

Additional supplementary materials:  crystallographic information; 3D view; checkCIF report

## Figures and Tables

**Table 1:** Hydrogen-bond geometry (Å, °)

*D*—H⋯*A*	*D*—H	H⋯*A*	*D*⋯*A*	*D*—H⋯*A*
O1*W*—H1*W*1⋯O1^i^	0.83 (2)	2.05 (2)	2.811 (3)	152 (4)
O1*W*—H1*W*2⋯O2^ii^	0.83 (2)	1.96 (2)	2.787 (3)	171 (5)
N1—H1*A*⋯O2^i^	0.91	1.84	2.745 (3)	177
N1—H1*C*⋯O1*W*	0.91	2.09	2.792 (4)	133
N1—H1*B*⋯O1^iii^	0.91	1.98	2.832 (3)	156

Symmetry codes: (i) 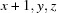; (ii) 
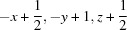
; (iii) 
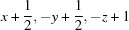
.

**Fig. 1.**
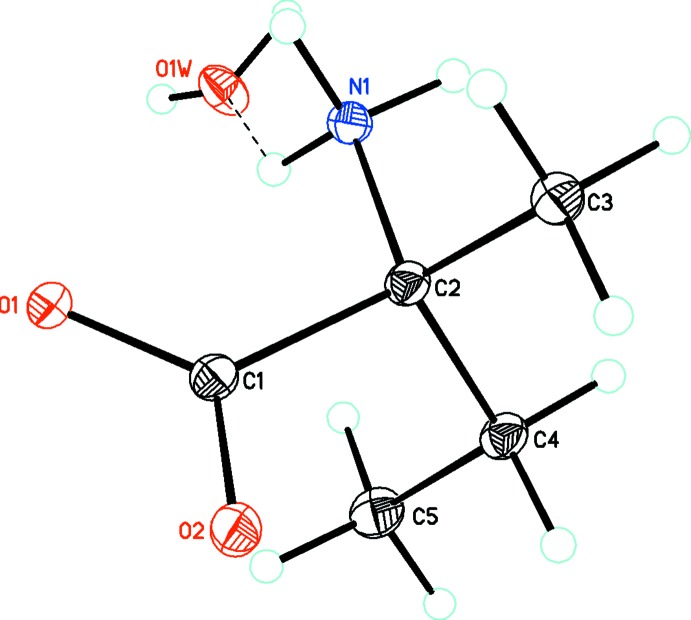
Diagram of the title compound showing atom labeling. Atomic displacement parameters are at the 30% probablity level. Hydogen bonds are shown as dashed lines.

**Fig. 2.**
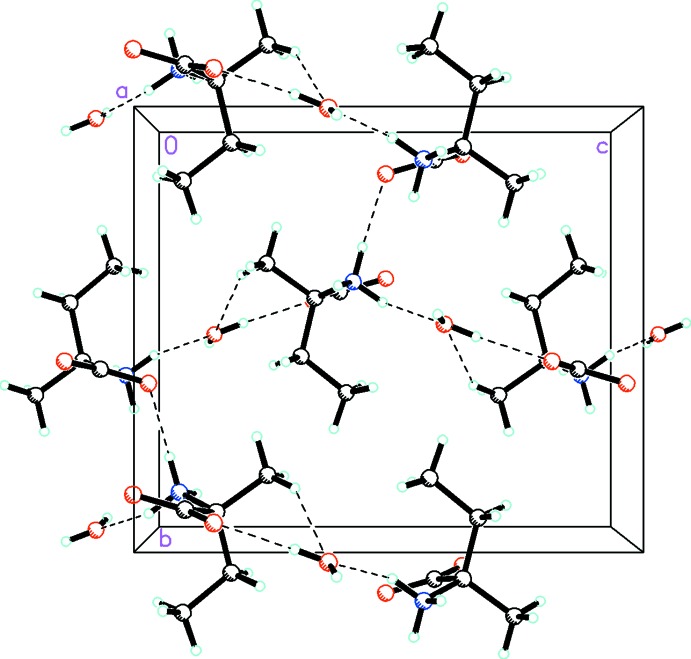
Packing diagram of the title compound viewed along the a axis showing tthe extensive N—H···O and O—H···O hydrogen bonds as dashed lines.

## supplementary crystallographic information

### 1. Comment

The alpha amino acids are essential for life as they are the building blocks of all proteins and enzymes. Nature uses almost exclusively the *L* form of the nineteen naturally occurring chiral amino acids. Glycine is achiral. However, it is known that there are over eighty amino acids that have been detected in meteorites or comets (Glavin & Dworkin 2009; Burton *et al.*, 2012). One of these extraterrestrial non proteinogenic amino acids is isovaline. The majority of these amino acids show little or no enrichment of one enantiomer over the other. An intriguing question is the process that lead to the separation and enrichment of the *L* enantiomer over the *D*. There are several possible explanations for this including the role that crystallization plays (Blackmond & Klussmann, 2007). Only two of the twenty amino acids used biologically crystallize in a chiral space group, which allows for spontaneous separation of enantiomers, at the level of the crystal, from a racemic solution (Blackmond *et al.*, 2008). Isovaline, a non proteinogenic amino acid also allows for this separation of enantiomers at the level of the crystal as it crystallizes in the chiral space group, *P*2_1_2_1_2_1_.

Another important aspect in the prebiotic chemistry of the amino acids is the role of racemization. All of the nineteen naturally occurring chiral amino acids have a hydrogen atom on the alpha carbon atom, which enhances the rate of racemization (Yamada *et al.*, 1983). However, little is known about the mechanism of racemization of amino acids lacking an alpha hydrogen atom (Pizzarello & Groy, 2011). The structure of isovaline given here can be used to examine the role that crystallization can play in the enrichment of *L* isovaline (Glavin & Dworkin 2009; Bada, 2009), and as a starting point in mechanistic studies of racemization mechanisms of isovaline (Bonner *et al.*, 1979).

In the structure of the title compound the amino acid is in the usual zwitterionic form. While the structure of the title compound or its salts have not been reported there are reports of homoleptic coordination complexes of isovaline with zinc(II) (Strasdelt *et al.* (2001). However, there are several important differences between these structures and the title compound. The metal complexes all crystallized in non-chiral space groups and, of course, when coordinated to a metal, the isovaline will not be in a zwitterionic form but, apart from the COO^-^ group, all the the bond lengths and angles are in the normal range for such compounds (Orpen, 1993). There is extensive N—H···O and O—H···O hydrogen bonding linking the zwitterions into a 3-D array.

### 2. Experimental

A sample of the title compound was obtained from Acros Organics. Crystals of the title compound were grown from the slow evaporation of a *racemic* solution of the amino acid in water.

### 3. Refinement

H atoms were placed in geometrically idealized positions and constrained to ride on their parent atoms with a C—H distances of 0.98 and 0.99 Å and an N—H distance of 0.91 \%A *U*_iso_(H) = 1.2*U*_eq_(C) and 0.96 Å for NH_3_ [*U*_iso_(H) = 1.5*U*_eq_(C)]. The water H's were refined isotropically with O—H distances constrained to be 0.82 Å and the H—O—H angle close to 104.5°.

### Crystal data

**Table:**

C_5_H_11_NO_2_·H_2_O	*F*(000) = 296
*M**_r_* = 135.16	*D*_x_ = 1.220 Mg m^−^^3^
Orthorhombic, *P*2_1_2_1_2_1_	Cu *K*α radiation, λ = 1.54184 Å
Hall symbol: P 2ac 2ab	Cell parameters from 679 reflections
*a* = 5.9089 (5) Å	θ = 3.7–75.3°
*b* = 10.4444 (10) Å	µ = 0.84 mm^−^^1^
*c* = 11.9274 (11) Å	*T* = 123 K
*V* = 736.10 (12) Å^3^	Needle, colorless
*Z* = 4	0.48 × 0.08 × 0.06 mm

### Data collection

**Table:**

Agilent Xcalibur (Ruby, Gemini) diffractometer	1204 independent reflections
Radiation source: Enhance (Cu) X-ray Source	1072 reflections with *I* > 2σ(*I*)
Graphite monochromator	*R*_int_ = 0.072
Detector resolution: 10.5081 pixels mm^-1^	θ_max_ = 75.4°, θ_min_ = 5.6°
ω scans	*h* = −7→4
Absorption correction: multi-scan (*CrysAlis PRO*; Agilent, 2012)	*k* = −12→8
*T*_min_ = 0.383, *T*_max_ = 1.000	*l* = −14→14
1662 measured reflections	

### Refinement

**Table:**

Refinement on *F*^2^	Primary atom site location: structure-invariant direct methods
Least-squares matrix: full	Secondary atom site location: difference Fourier map
*R*[*F*^2^ > 2σ(*F*^2^)] = 0.056	Hydrogen site location: inferred from neighbouring sites
*wR*(*F*^2^) = 0.162	H atoms treated by a mixture of independent and constrained refinement
*S* = 1.11	*w* = 1/[σ^2^(*F*_o_^2^) + (0.093*P*)^2^ + 0.2964*P*] where *P* = (*F*_o_^2^ + 2*F*_c_^2^)/3
1204 reflections	(Δ/σ)_max_ < 0.001
91 parameters	Δρ_max_ = 0.37 e Å^−^^3^
3 restraints	Δρ_min_ = −0.27 e Å^−^^3^

### Special details

**Table:**

Geometry. All e.s.d.'s (except the e.s.d. in the dihedral angle between two l.s. planes) are estimated using the full covariance matrix. The cell e.s.d.'s are taken into account individually in the estimation of e.s.d.'s in distances, angles and torsion angles; correlations between e.s.d.'s in cell parameters are only used when they are defined by crystal symmetry. An approximate (isotropic) treatment of cell e.s.d.'s is used for estimating e.s.d.'s involving l.s. planes.
Refinement. Refinement of *F*^2^ against ALL reflections. The weighted *R*-factor *wR* and goodness of fit *S* are based on *F*^2^, conventional *R*-factors *R* are based on *F*, with *F* set to zero for negative *F*^2^. The threshold expression of *F*^2^ > σ(*F*^2^) is used only for calculating *R*-factors(gt) *etc*. and is not relevant to the choice of reflections for refinement. *R*-factors based on *F*^2^ are statistically about twice as large as those based on *F*, and *R*- factors based on ALL data will be even larger.

### Fractional atomic coordinates and isotropic or equivalent isotropic displacement parameters (Å^2^)

**Table:**

	*x*	*y*	*z*	*U*_iso_*/*U*_eq_	
O1	0.0058 (4)	0.37096 (19)	0.50201 (18)	0.0316 (5)	
O2	−0.1536 (4)	0.4257 (3)	0.33811 (19)	0.0385 (6)	
O1W	0.6455 (4)	0.4874 (3)	0.6172 (2)	0.0467 (7)	
H1W1	0.776 (5)	0.465 (5)	0.601 (3)	0.070*	
H1W2	0.661 (8)	0.517 (5)	0.682 (2)	0.070*	
N1	0.4267 (4)	0.3850 (2)	0.4302 (2)	0.0297 (6)	
H1A	0.5663	0.3957	0.3994	0.045*	
H1B	0.4074	0.3014	0.4493	0.045*	
H1C	0.4141	0.4348	0.4925	0.045*	
C1	0.0133 (6)	0.4049 (2)	0.4006 (3)	0.0302 (6)	
C2	0.2504 (5)	0.4229 (3)	0.3472 (2)	0.0294 (6)	
C3	0.2766 (6)	0.3382 (3)	0.2440 (3)	0.0367 (7)	
H3A	0.2503	0.2486	0.2648	0.055*	
H3B	0.4301	0.3474	0.2139	0.055*	
H3C	0.1664	0.3640	0.1869	0.055*	
C4	0.2832 (6)	0.5657 (3)	0.3178 (3)	0.0344 (7)	
H4A	0.4384	0.5779	0.2885	0.041*	
H4B	0.1762	0.5888	0.2572	0.041*	
C5	0.2477 (7)	0.6556 (3)	0.4151 (3)	0.0451 (9)	
H5A	0.2565	0.7442	0.3886	0.068*	
H5B	0.3650	0.6408	0.4716	0.068*	
H5C	0.0984	0.6401	0.4482	0.068*	

### Atomic displacement parameters (Å^2^)

**Table:**

	*U*^11^	*U*^22^	*U*^33^	*U*^12^	*U*^13^	*U*^23^
O1	0.0326 (11)	0.0293 (9)	0.0329 (10)	0.0009 (8)	0.0031 (9)	0.0061 (9)
O2	0.0268 (11)	0.0510 (13)	0.0376 (11)	0.0010 (11)	0.0007 (9)	0.0110 (11)
O1W	0.0379 (13)	0.0627 (17)	0.0396 (13)	0.0087 (13)	−0.0008 (11)	−0.0148 (12)
N1	0.0268 (12)	0.0270 (11)	0.0352 (14)	−0.0004 (9)	0.0016 (11)	0.0015 (11)
C1	0.0324 (15)	0.0214 (12)	0.0367 (15)	−0.0017 (11)	0.0027 (12)	0.0020 (11)
C2	0.0279 (15)	0.0275 (13)	0.0328 (14)	−0.0009 (12)	0.0009 (11)	0.0051 (13)
C3	0.0371 (18)	0.0392 (16)	0.0338 (16)	0.0040 (15)	−0.0005 (13)	0.0010 (14)
C4	0.0320 (15)	0.0317 (15)	0.0394 (15)	−0.0038 (13)	−0.0001 (13)	0.0085 (14)
C5	0.055 (2)	0.0260 (14)	0.054 (2)	−0.0030 (14)	0.0026 (18)	0.0020 (15)

### Geometric parameters (Å, º)

**Table:**

O1—C1	1.261 (4)	C2—C4	1.545 (4)
O2—C1	1.255 (4)	C3—H3A	0.9800
O1W—H1W1	0.830 (19)	C3—H3B	0.9800
O1W—H1W2	0.832 (19)	C3—H3C	0.9800
N1—C2	1.490 (4)	C4—C5	1.507 (5)
N1—H1A	0.9100	C4—H4A	0.9900
N1—H1B	0.9100	C4—H4B	0.9900
N1—H1C	0.9100	C5—H5A	0.9800
C1—C2	1.550 (4)	C5—H5B	0.9800
C2—C3	1.524 (4)	C5—H5C	0.9800
			
H1W1—O1W—H1W2	102 (3)	C2—C3—H3B	109.5
C2—N1—H1A	109.5	H3A—C3—H3B	109.5
C2—N1—H1B	109.5	C2—C3—H3C	109.5
H1A—N1—H1B	109.5	H3A—C3—H3C	109.5
C2—N1—H1C	109.5	H3B—C3—H3C	109.5
H1A—N1—H1C	109.5	C5—C4—C2	114.2 (3)
H1B—N1—H1C	109.5	C5—C4—H4A	108.7
O2—C1—O1	126.2 (3)	C2—C4—H4A	108.7
O2—C1—C2	116.4 (3)	C5—C4—H4B	108.7
O1—C1—C2	117.4 (3)	C2—C4—H4B	108.7
N1—C2—C3	108.1 (3)	H4A—C4—H4B	107.6
N1—C2—C4	108.6 (3)	C4—C5—H5A	109.5
C3—C2—C4	111.3 (3)	C4—C5—H5B	109.5
N1—C2—C1	109.1 (2)	H5A—C5—H5B	109.5
C3—C2—C1	110.7 (3)	C4—C5—H5C	109.5
C4—C2—C1	108.9 (2)	H5A—C5—H5C	109.5
C2—C3—H3A	109.5	H5B—C5—H5C	109.5
			
O2—C1—C2—N1	−175.8 (3)	O1—C1—C2—C4	−114.2 (3)
O1—C1—C2—N1	4.2 (4)	N1—C2—C4—C5	−64.0 (3)
O2—C1—C2—C3	−57.0 (3)	C3—C2—C4—C5	177.0 (3)
O1—C1—C2—C3	123.0 (3)	C1—C2—C4—C5	54.7 (4)
O2—C1—C2—C4	65.8 (3)		

### Hydrogen-bond geometry (Å, º)

**Table:**

*D*—H···*A*	*D*—H	H···*A*	*D*···*A*	*D*—H···*A*
O1*W*—H1*W*1···O1^i^	0.83 (2)	2.05 (2)	2.811 (3)	152 (4)
O1*W*—H1*W*2···O2^ii^	0.83 (2)	1.96 (2)	2.787 (3)	171 (5)
N1—H1*A*···O2^i^	0.91	1.84	2.745 (3)	177
N1—H1*C*···O1*W*	0.91	2.09	2.792 (4)	133
N1—H1*B*···O1^iii^	0.91	1.98	2.832 (3)	156

Symmetry codes: (i) *x*+1, *y*, *z*; (ii) −*x*+1/2, −*y*+1, *z*+1/2; (iii) *x*+1/2, −*y*+1/2, −*z*+1.

## References

[bb1] Agilent (2012). *CrysAlis PRO* Agilent Technologies UK Ltd, Yarnton, England.

[bb2] Bada, J. L. (2009). *Proc. Natl Acad. Sci.* **106**, E85.

[bb3] Blackmond, D. G. & Klussmann, M. (2007). *Chem. Commun.* pp. 3990–3996. 10.1039/b709314b 17912393

[bb4] Blackmond, D., Viedma, C., Ortiz, J., Torres, T. & Izuma, T. (2008). *J. Am. Chem. Soc.* **130**, 15274–15275. 10.1021/ja8074506 18954052

[bb5] Bonner, W. A., Blair, N. E., Lemmon, R. M., Flores, J. J. & Pollock, G. E. (1979). *Geochim. Cosmochim. Acta*, **43**, 1841–1846.

[bb6] Burton, A. S., Stern, J. C., Elsila, J. E., Dworkin, J. P. & Glavin, D. P. (2012). *Chem. Soc. Rev.* **41**, 5459–5472. 10.1039/c2cs35109a 22706603

[bb8] Glavin, D. P. & Dworkin, J. P. (2009). *Proc. Natl Acad. Sci.* **106**, 5487–5492. 10.1073/pnas.0811618106 PMC2667035 19289826

[bb9] Orpen, G. A. (1993). *Chem. Soc. Rev.* **22**, 191–197.

[bb10] Pizzarello, S. & Groy, T. L. (2011). *Geochim. Cosmochim. Acta*, **75**, 645–656.

[bb11] Sheldrick, G. M. (2008). *Acta Cryst.* A**64**, 112–122. 10.1107/S0108767307043930 18156677

[bb12] Strasdelt, H., Busching, I., Behrends, S., Saak, W. & Barklage, W. (2001). *Chem. Eur. J.* **7**, 1133–1137. 10.1002/1521-3765(20010302)7:5<1133::aid-chem1133>3.0.co;2-t 11303873

[bb13] Yamada, S., Hongo, C., Yoshioka, R. & Chibata, I. (1983). *J. Org. Chem.* **48**, 843–846.

